# Upregulation of CPE promotes cell proliferation and tumorigenicity in colorectal cancer

**DOI:** 10.1186/1471-2407-13-412

**Published:** 2013-09-05

**Authors:** Xing-Hua Liang, Ling-ling Li, Geng-Gang Wu, Yi-Cheng Xie, Guang-Xian Zhang, Wei Chen, Hai-Feng Yang, Qi-Long Liu, Wen-Hong Li, Wen-guang He, Yan-Nian Huang, Xian-Cheng Zeng

**Affiliations:** 1 Department of Gastroenterology, Zengcheng People’s Hospital, (BoJi-Affiliated Hospital of Sun Yat-Sen University), Zengcheng 511300, China; 2 Central Laboratory, The Fifth Affiliated Hospital of Sun Yat-sen University, Zhuhai 519000, China; 3 Department of General Surgery, Zengcheng People’s Hospital, (BoJi-Affiliated Hospital of Sun Yat-Sen University), Zengcheng 511300, China; 4 School of Basic Medical Sciences, Guangzhou University of Chinese Medicine, Guangzhou 510006, China; 5 Department of Hepatopancreatobiliary Surgery, Second Affiliated Hospital, School of Medicine, Zhejiang University, Hangzhou 310009, China; 6 Department of Pathology, The Second Affiliated Hospital of Guangzhou University of Chinese Medicine (Guangdong Provincial Hospital of TCM), Guangzhou 510120, China; 7 Department of Clinical Laboratory, Zengcheng People’s Hospital, (BoJi-Affiliated Hospital of Sun Yat-Sen University), Zengcheng 511300, China

**Keywords:** Colorectal cancer, CPE, Cell proliferation, Tumorigenicity

## Abstract

**Background:**

Colorectal cancer (CRC) is one of the most common cancers worldwide and a leading cause of cancer related death. Although the mortality rate of CRC is decreasing, finding novel targets for its therapy remains urgent. Carboxypeptidase E (CPE), a member of the pro-protein convertases, which are involved in the maturation of protein precursors, has recently been reported as elevated in many types of cancer. However, its role and mechanisms in tumor progression are poorly understood.

**Methods:**

In the present study, we investigated expression of CPE in CRC cell lines and tumor tissues using Western blot and real-time qRT-PCR. Plasmids for overexpression and depletion of CPE were constructed and analyzed by Western blot, MTT and colony formation assays and bromodeoxyuridine incorporation assays. The relative expression of p21, p27, and cyclin D1 were analyzed by Real-time qRT-PCR in the indicated cells.

**Results:**

Our study showed that CPE was significantly upregulated in CRC cell lines and tumor tissues. MTT and colony formation assays indicated that overexpression of CPE enhanced cell growth rates. BrdU incorporation and flow-cytometry assays showed that ectopic expression of CPE increased the S-phase fraction cells. Soft agar assay proved enhanced tumorigenicity activity in CPE over-expressing CRC cells. Further studies of the molecular mechanisms of CPE indicated that is promoted cell proliferation and tumorigenicity through downregulation of p21 and p27, and upregulation of cyclin D1.

**Conclusions:**

Taken together, these data suggest that CPE plays an important role in cell cycle regulation and tumorigenicity, and may serve as a potential target for CRC therapeutics.

## Background

Colorectal cancer (CRC) is one of the most common cancers and a leading cause of cancer-related deaths [1]. According to WHO statistics, there were an estimated 1.2 million cases of CRC in 2008 worldwide. CRC development is a multi-step and multigene process, involving activation and overexpression of oncogenes, and inactivation and downregulation of tumor suppressor genes [2], which have multiple effects in CRC tumorigenesis, including cell proliferation, apoptosis, invasion, and metastasis. Mutation of the tumor suppresser gene, adenomatous polyposis coli (APC), is one of the most well studied in CRC. Familial adenomatous polyposis (FAP), an autosomal, dominantly inherited disease, can cause the development of hundreds to thousands of colorectal tumors during the second and third decades of a patient’s life [3]. Germline mutations in APC are identified in approximately 80% of FAP affected individuals [4,5]. Many other oncoproteins have been reported to be upregulated or activated in CRC, such as FOXQ1 [6], PIK3CA [7], and cyclin D1 [8]. Although CPE, a prohormone/proneuropeptide processing enzyme, has been reported to be elevated in CRC [9], its role in tumor development remains poorly understood.

CPE is found primarily in endocrine and neuroendocrine cells, and is a metalloexopeptidase [10]. It encodes a carboxypeptidase that cleaves C-terminal amino acid residues, and is involved in the biosynthesis of peptide hormones and neuropeptides, which are synthesized as precursors in the rough endoplasmic reticulum. After being packaged into secretory granules, these precursors are processed sequentially, first, by prohormone convertases (PC1/3 and PC2) to remove the carboxyl side of paired basic residues to yield basic residue-extended peptides [11,12], then by a subset of soluble CPE to cleave the basic residues to generate biologically active peptide hormones and neuropeptides [10,13,14]. CPE also functions as a prohormone sorting receptor for the regulated secretory pathway (RSP) [15,16]. Mice with Cpe mutations, or Cpe knockout mice, exhibit pathophysiological conditions, such as obesity, diabetes, infertility, low bone mineral density, and deficits in learning and memory [17-20]. In humans, deregulated CPE has been associated with numerous diseases, such as diabetes, Alzheimer's disease [21], and cancers. Horing *et al*. found reduced expression of CPE in Glioblastoma, and proposed that CPE functioned as a putative tumor suppressor gene [22]. Conversely, Murthy *et al*. reported that CPE was significantly elevated in many human cancers, and its upregulation was correlated with tumor growth and metastasis [9]. Therefore, the role of CPE in cancer remains unclear.

In this study, we found that CPE was significantly upregulated in CRC cell lines and tumor tissues. Further investigations revealed that overexpression of CPE led to decreased expression of cyclin-dependent kinase (CDK) inhibitors, p21 and p27, and increased expression of the CDK regulator, cyclin D1. The resulting increase in the S-phase fraction of tumor cells may account for CPE’s role in enhancing cell growth rates and tumorigenicity activity in CRC cells. These results suggest that CPE may be a novel target for CRC therapeutics.

## Methods

### Ethics statement

For the use of clinical materials for research purposes, samples were obtained with prior written informed consents from the patients and approval from the Institutional Research Ethics Committees of ZengchengPeople^,^s Hospital (BoJi-Affiliated Hospital of Sun Yat-Sen University) ethics Committee.

### Cell lines and tissue specimens

Colorectal cancer (CRC) cell lines, including SW480, SW620, KM12, HCT15, HCT116, Caco-2, and LoVo, were cultured in RPMI 1640 medium (Invitrogen, Carlsbad, CA, US) supplemented with 10% FBS (HyClone, Logan, Utah, US). Tissue specimens were freshly collected from Zengcheng People’s Hospital (BoJi-Affiliated Hospital of Sun Yat-Sen University), and were histopathologically and clinically diagnosed.

### Plasmids and antibodies

For overexpression of CPE: human full-length CPE cDNA from HCT116 cells was amplified by PCR and cloned into a pMSCV-puro retroviral vector. For depletion of CPE: two human shRNA sequences were cloned into the pSuper-retro-puro plasmid to generate pSuper-retro CPE shRNA. The following sequences were selected: RNAi#1, CTCCAGGCTATCTGGCAATAA; and RNAi#2, GATAGGATAGTGTACGTGAAT. Anti-CPE (BD, Franklin Lakes, New Jersey, US), anti-β-actin (Sigma, Saint Louis, MI, US), and anti-BrdU (Upstate, Temecula, CA, US) were used for Western blot analysis and bromodeoxyuridine (BrdU) incorporation assays.

### RNA extraction, reverse transcription (RT) and real-time qRT-PCR

Total RNA from cultured cells was extracted using Trizol reagent (Invitrogen, Carlsbad, CA, US), following the manufacturer’s instructions. The cDNA was amplified and quantified using an ABI Prism 7500 Sequence Detection System (Applied Biosystems, Foster City, CA), with SYBR Green I dye (Molecular Probes, Invitrogen, CA Carlsbad, CA, US). The following primers were selected: CPE: CCATCAGCAGGATTTACACG (forward) and TAAATTCAGGCTCACCAGGC (reverse); p21: CGATGCCAACCTCCTCAACGA (forward) and TCGCAGACCTCCAGCATCCA (reverse); p27: TGCAACCGAC GATTCTTCTACTCAA (forward) and CAAGCAGTGATGTATCTGATAAACA AGGA (reverse); Cyclin D1: AACTACCTGGACCGCTTCCT (forward) and CCAC TTGAGCTTGTTCACCA (reverse); GAPDH: ACCACAGTCCATGCCATCAC (forward) and TCCACCACCCTGTTGCTGTA (reverse). Expression data were normalized to GAPDH, and calculated as 2^-(*C*^*t*^[gene] – *C*^*t*^[*GAPDH*])^, where C_t_ represents the threshold cycle for each transcript.

### MTT and colony formation assay

For the MTT assay: cells were seeded in 96-well plates (2000 cells/plate); at each time point, cells were stained with 100 μl sterile MTT dye (0.5 mg/ml; Sigma, St. Louis, MO, US) for 4 h at 37°C; the culture medium was removed; and 150 μl of dimethyl sulphoxide (DMSO; Sigma) was added. The absorbance was measured at 570 nm; the reference wavelength was 655 nm. For the colony formation assay: cells were seeded in 6-well plates (1000 cells/plate); cultured for 10 days; fixed with ice-cold methanol for 10 min; and stained with 1% crystal violet for 1 min.

### Bromodeoxyuridine incorporation assay

Cells were seeded on coverslips (Fisher, Pittsburgh, PA, US) in 24-well plates (5 × 10^4^ cells/plate). After 24 h, the cells were incubated with BrdU for 1 h, and stained with anti-BrdU antibody (Upstate, Temecula, CA, US), following the manufacturer’s instructions. Gray level images were acquired under a laser scanning microscope (Axioskop 2 plus, Carl Zeiss Co. Ltd., Jena, Germany).

### Anchorage-independent growth ability assay

Cells (5000 cells/plate) were mixed in 2 × RPMI 1640 with an equal volume of soft agar (Sigma, Saint Louis, MO, US) to give a final solution of 0.3% agar, 1 × RPMI 1640, 10% FBS. The cell-agar mixture was added to the top of the cell-free bottom layer with 1% agar. After 10 days, viable colonies larger than 0.1 mm were counted.

### Statistical analysis

Statistical tests for data analyses are Student’s 2-tailed t test. Statistical analyses were performed using the SPSS 11.0 statistical software package. Data represent mean ± SD. *P* values of 0.05 or less were considered statistically significant.

## Results

### CPE is overexpressed in CRC cell lines and tissues

**Figure 1:**
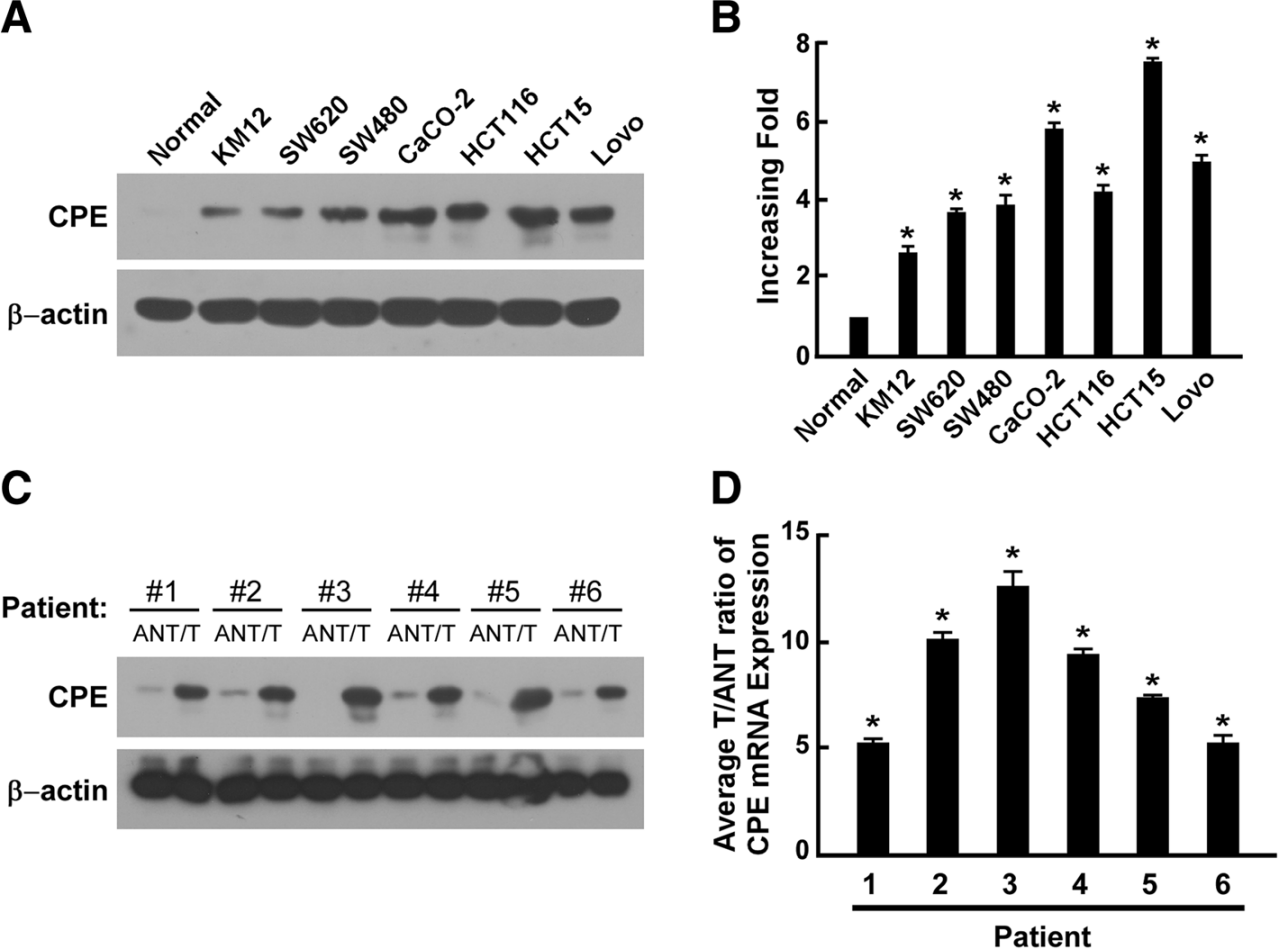
**CPE is overexpressed in colorectal cancer cells. (A**-**B)** Western blot analysis **(A)** andreal-time qRT-PCR analysis **(B)** showing the relative expression of CPE in CRC cell lines and primary normal colorectal epithelial cells. **(C**-**D)** Western blot analysis **(C)** and real-time qRT-PCR analysis **(D)** showing the relative expression of CPE in CRC patients’ tumor tissues (T) vs. matched adjacent normal tissues (ANT); β-actin was used as a loading control. mRNA data were normalized to *GAPDH* control and are presented as mean ± standard deviation (SD) from three independent experiments. *: *P <* 0.05.

To investigate the biological role of CPE in human CRC progression, we analyzed CPE expression in CRC cell lines and paired tissue specimens from CRC patients. Western blot analysis and real-time qRT-PCR results showed that CPE was overexpressed in all CRC cell lines compared to primary normal colorectal epithelial cells (Figure 1A-B). Data from paired CRC tissue specimens showed that both CPE protein and mRNA were significantly upregulated (5- to 13-fold) in tumor tissue compared to matched adjacent normal tissue (Figure 1C-D and Additional file 1: Figure S1). Taken together, these data indicated that CPE was overexpressed in CRC, and its overexpression may contribute to the development of human CRC.

### CPE expression levels correlate with cell proliferation rates in CRC

**Figure 2:**
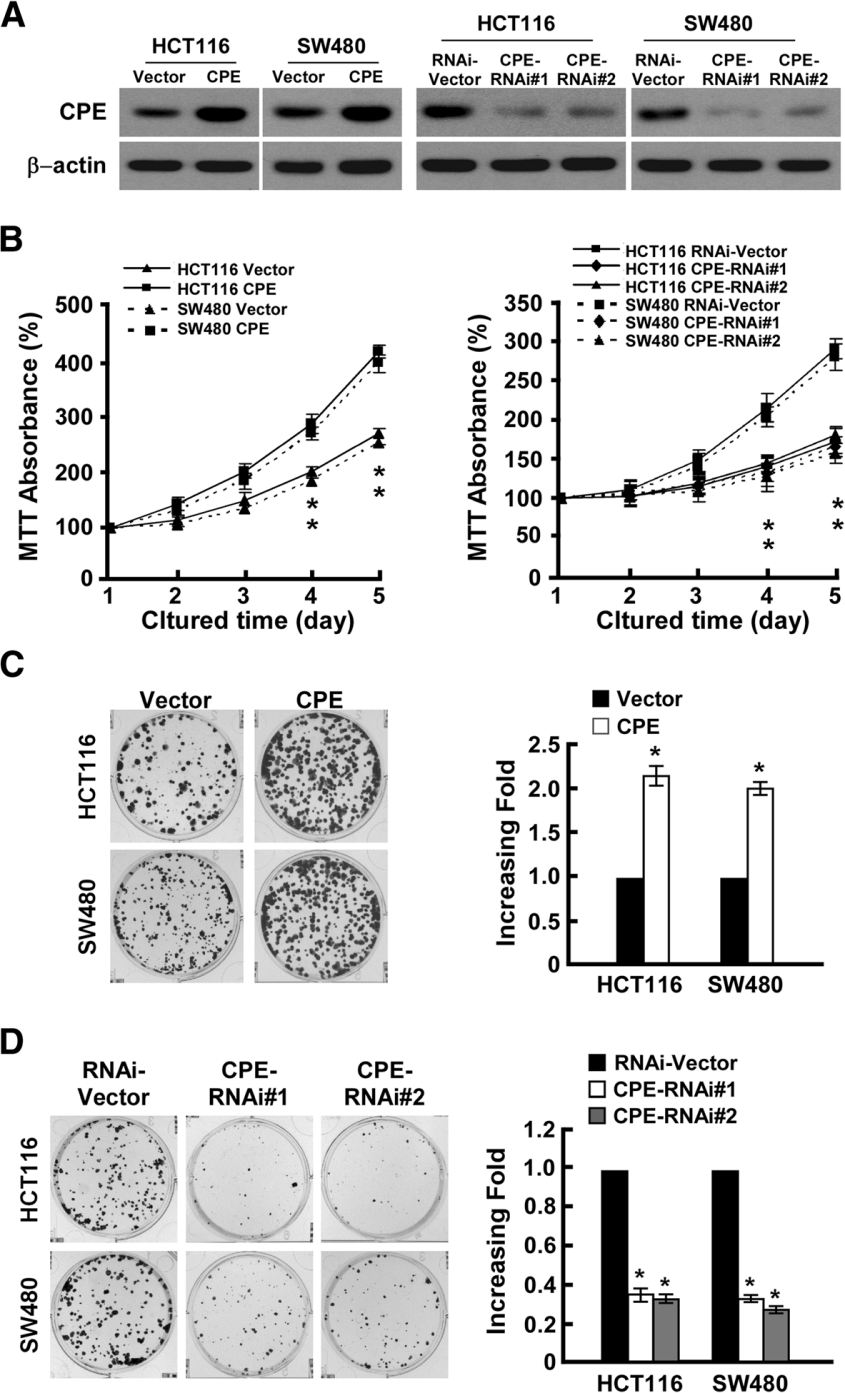
**CPE promotes colorectal cancer cell proliferation. (A)** Western blot analysis of CPE expression in HCT116 and SW480 cell lines stably infected with CPE ORF or shRNA. β-actin was used as a loading control. **(B)** MTT assay analysis of cell growth rates for different stable cell lines at the indicated times after seeding cells. **(C)** Representative micrographs (left panel) and quantification (right panel) of colony formation in CPE-overexpressing and vector cells. **(D)** Representative micrographs (left panel) and quantification (right panel) of colony formation in CPE-silencing and vector cells. Data are presented as mean ± SD from three independent experiments. *: *P <* 0.05.

To further investigate the role of CPE in CRC, two CRC cell lines, HCT116 and SW480, were selected to stably express CPE ORF and CPE shRNA. Western blot analysis showed that stable cell lines were successfully established (Figure 2A). The role of CPE in cell proliferation was investigated by conducting MTT and colony formation assays. Ectopic expression of CPE dramatically enhanced growth rates of both CRC cell lines (Figure 2B, left panel), forming more and larger colonies (Figure 2C). Conversely, silencing of CPE impaired growth rates (Figure 2B, right panel) and colony formation abilities (Figure 2D) in both CRC cell lines. Herein, we concluded that overexpression of CPE promotes CRC cell proliferation.

### CPE promotes cell proliferation by increasing the S-phase fraction of CRC cells

**Figure 3:**
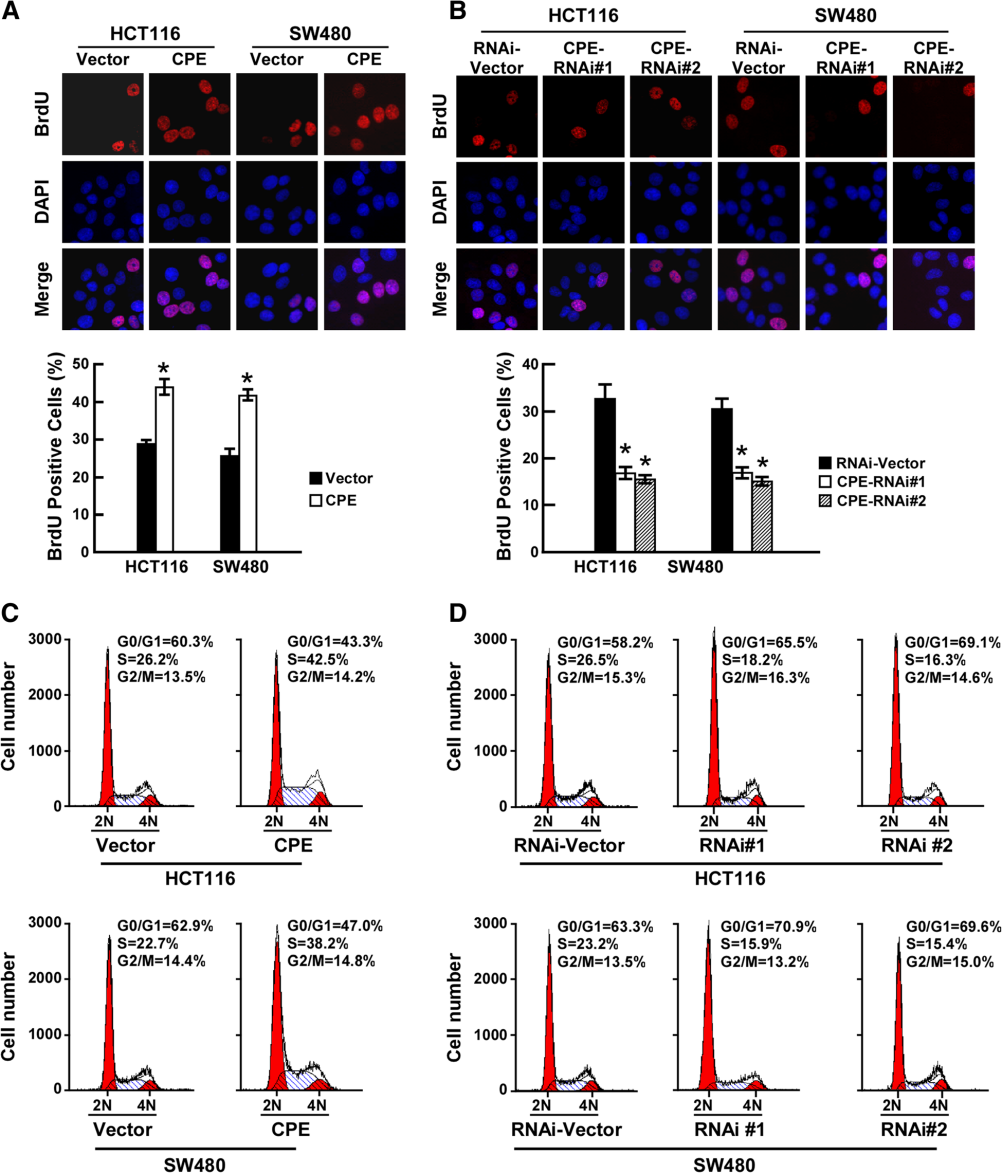
**CPE promotes cell proliferation by increasing the S-phase fraction of cells. (A)** Representative micrographs (upper panel) and quantification (lower panel) of BrdU incorporation in CPE-overexpressing and vector cells. **(B)** Representative micrographs (upper panel) and quantification (lower panel) of BrdU incorporation in CPE-silencing and vector cells. **(C)** Flow cytometric analysis of CPE-overexpressing and vector cells. **(D)** Flow cytometric analysis of CPE-silencing and vector cells. Data are presented as mean ± SD from three independent experiments. *: *P <* 0.05.

Having observed that CPE upregulation promoted cell proliferation, we further explored the underling mechanisms. BrdU, an analogue of thymidine, becomes incorporated into replicating DNA by replacing thymidine. Subsequent immunodetection of BrdU allows the percent-age of cells at S-phase to be determined. As shown in Figure 3A (right panel), overexpression of CPE significantly increased the percentage of BrdU positive cells in both cell lines: 29.2% vs. 43.28% in HCT116; 26.88% vs. 41.36% in SW480. In contrast, knockdown of CPE dramatically decreased the S-phase fraction of BrdU incorporated cells: from 32.33% to 17.28% (CPE-RNAi#1) and 15.69% (CPE-RNAi#2) in HCT116 cells; and from 30.08% to 18.02% (CPE-RNAi#1) and 14.89% (CPE-RNAi#2) in SW480 cells (Figure 3B). Cell cycle analysis by flow-cytometry assay further proved that upregulation of CPE dramatically increased the percentage of S phase cells and decreased the percentage of cells in the G1/G0 phase (Figure 3C). Conversely, silencing of CPE increased the percentage of cells in the G1/G0 phase and decreased the percentage of S-phase cells (Figure 3D). Based on these data, we proposed that CPE promotes cell proliferation by increasing the S-phase fraction of CRC cells.

### Overexpression of CPE promotes tumorigenicity of CRC cells

**Figure 4:**
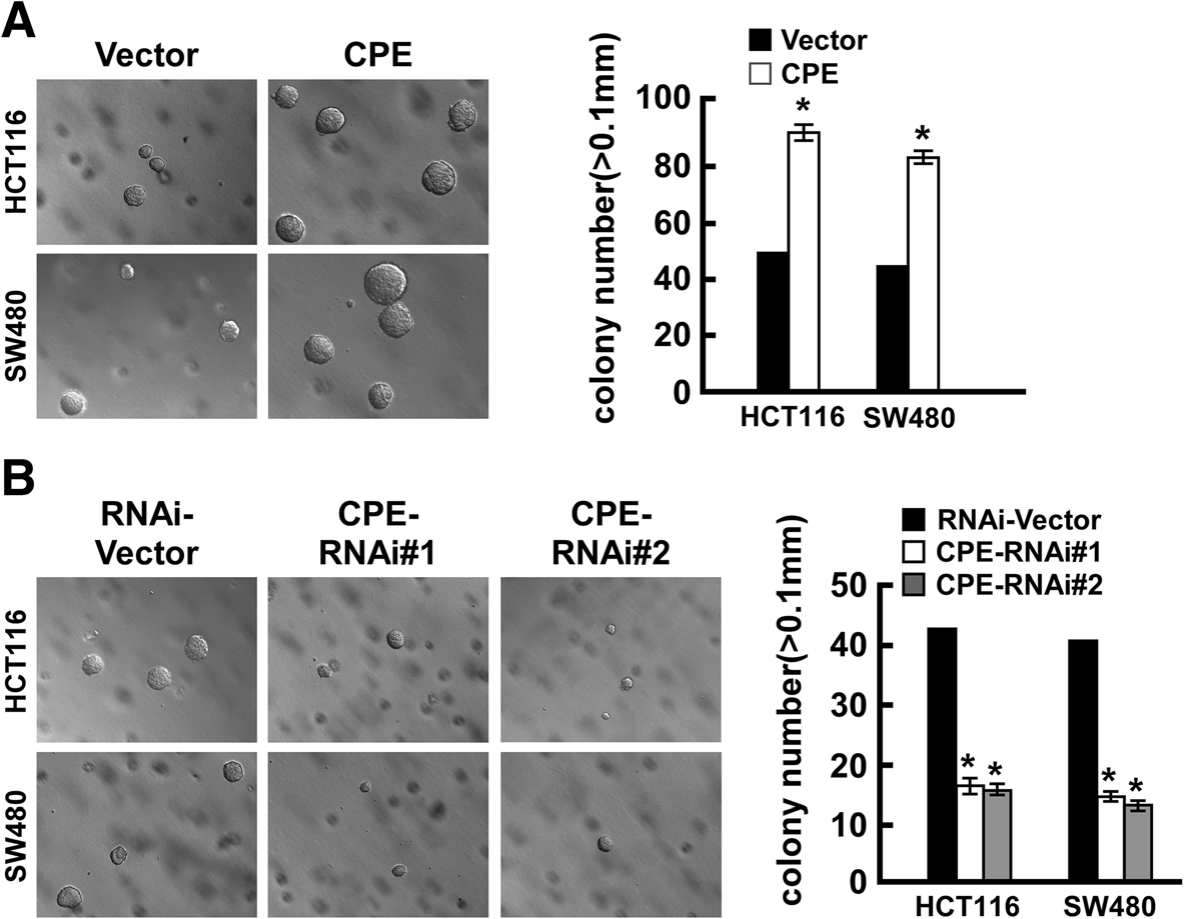
**Overexpression/knockdown of CPE promotes/impairs tumorigenicity of colorectal cancer cells. (A)** Representative micrographs (left panel) and quantification (right panel) of colonies formed in soft agar in CPE-overexpressing and vector cells. **(B)** Representative micrographs (left panel) and quantification (right panel) of colonies formed in soft agar in CPE-silencing and vector cells. Data are presented as mean ± SD from three independent experiments. *: *P <* 0.05.

To investigate the role of CPE expression on the tumorigenic activity of CRC cells, anchorage-independent growth ability assay was performed. The results showed that ectopic expression of CPE significantly enhanced anchorage-independent growth of both CRC cell lines, increasing the numbers and size of colonies in soft agar compared to vector cells (Figure 4A). Depletion of CPE dramatically impaired the anchorage-independent growth of both cell lines, as indicated by the reduction in colony numbers and colony size (Figure 4B).

### CPE promotes cell proliferation and tumorigenicity via modulation of p21 and p27 and cyclin D1 expression

**Figure 5:**
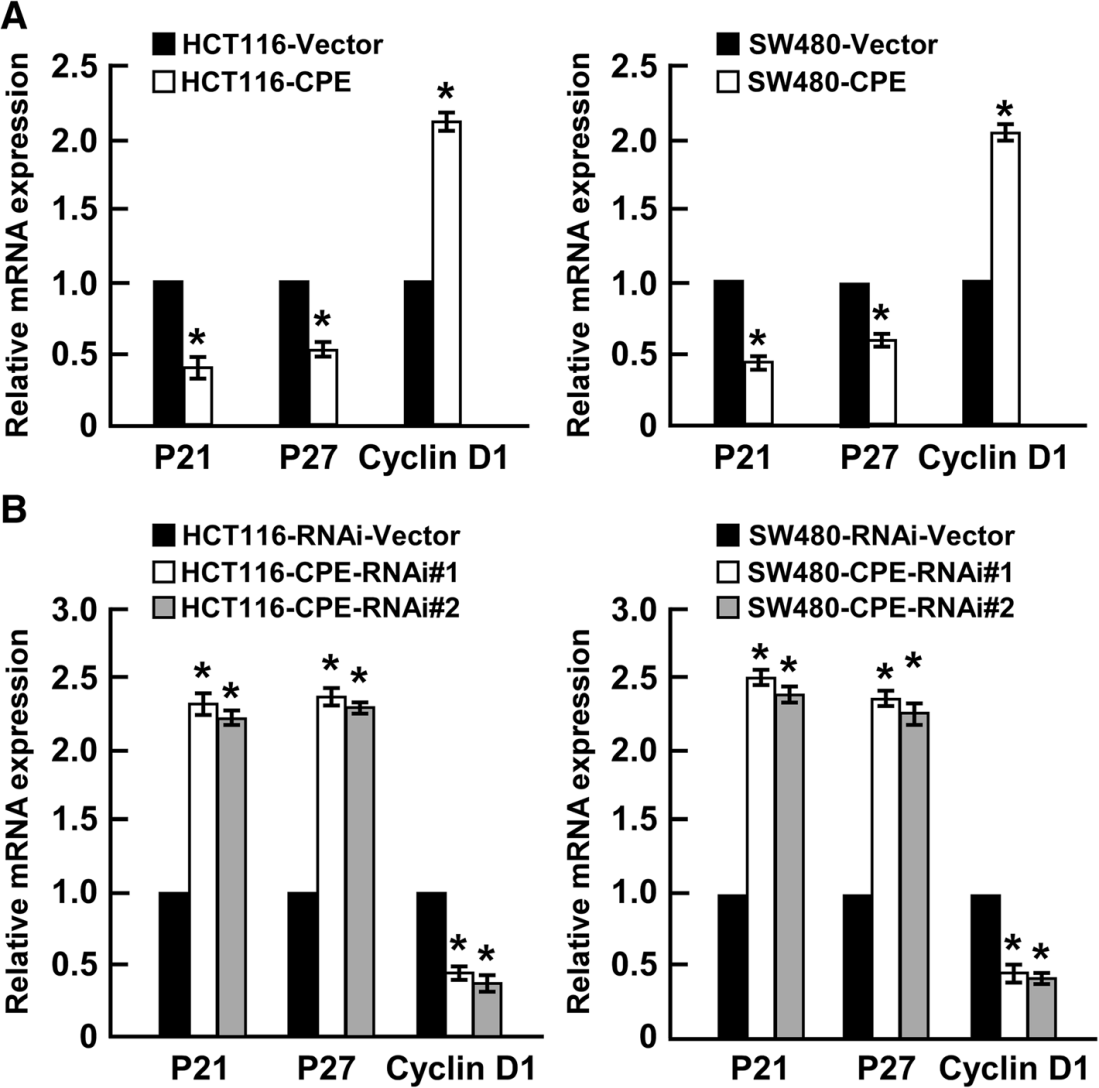
**CPE regulates p21, p27, and cyclin D1 expression. (A)** Overexpression of CPE downregulated p21 and p27, and upregulated cyclin D1. Real-time qRT-PCR analysis of the relative expression of p21, p27, and cyclin D1 in the indicated cells. **(B)** Knockdown of CPE upregulated p21 and p27, and downregulated cyclin D1. Real-time qRT-PCR analysis of the relative expression of p21, p27, and cyclin D1 in the indicated cells. Data were normalized to *GAPDH* control and presented as mean ± SD from three independent experiments. *: *P <* 0.05.

The CDK inhibitors p21 and p27, and CDK regulator cyclin D1, perform important functions in the control of cell cycle progression. Quantitive real-time PCR showed that overexpression of CPE significantly downregulated p21 and p27, and upregulated cyclin D1 (Figure 5A). In contrast, silencing of CPE dramatically enhanced p21 and p27 expression, and inhibited cyclin D1 expression in both HCT116 and SW480 cell lines (Figure 5B). These results indicated that CPE regulates p21, p27, and cyclin D1 to promote cell proliferation and tumorigenicity.

## Discussion and conclusion

In the present study, we found that CPE is elevated in CRC and have suggested a mechanistic role for CPE in the proliferation of CRC cell lines. Furthermore, we proposed that CPE possesses oncogenic functions in CRC development. This is consistent with other studies: Lee *et al*. reported that an N-terminal truncated CPE isoform was highly upregulated, and could induce tumor growth. They further suggested its use as a biomarker for predicting metastasis in hepatocellular carcinoma [23]. By analyzing profile data in the Gene Expression Omnibus   (GEO) database  (http://www.ncbi.nlm.nih.gov/geo/), Murthy *et al*. found that CPE was elevated in CRC and many other types of human cancer, including hepato-cellular carcinoma, cervical cancer, and kidney cancer [9]. However, in glioma, CPE expression level appears to be controversial: Liu *et al*. analyzed 12 primary brain glioma biopsies using cDNA microarrays, and revealed elevation of CPE expression compared to normal brain tissue [24]; this was supported by another cDNA microarray study which found elevation of CPE expression in 50 human gliomas of various histogenesis, compared to normal brain tissue samples [9]. In contrast, a study by Horing *et al*. indicated that CPE acted as a tumor suppressor by reducing expression of CPE in a cell death-resistant glioma cell line, and in GBM samples from The Cancer Genome Atlas cohort, compared to normal control brain specimens [22]. Therefore, regulation of CPE may be different in different types of cancer, and it is not yet possible to define CPE as either a tumor suppressor or an oncogene.

CPE is known to be elevated in many types of human cancer, irrespective of whether they are neuroendocrine tumors, such as lung cancer [25], or nonendocrine can-cers, such as CRC, as shown by our data and others (Saravana, [9]). However, the mechanism of CPE upregulation is still unknown. CPE locates at 4q32.3 in the human genome; and duplications of 4q31-qter have been documented in several human diseases [26-28], which may contribute to CPE amplification in cancer. Similarly, CPE upregulation has been reported in cervical cancer, where 70% of cases are correlated with human papillomavirus (HPV) infection [29], suggesting that CPE upregulation may triggered by viral antigens.

CPE is reported primarily in endocrine and neuroendocrine cells; however, it has now been identified in epithelial-derived cancer cells. CPE functions as a prohormone and neuropeptide processing exopeptidase, and as a regulated secretory pathway (RSP) sorting receptor [13-15]. Consequently, it has important roles in the endocrine and neural systems. In mice, mutation or knockout (KO) of CPE causes deficiencies in peptide hormones and neuropeptides, such as insulin [17,19], gonadotropin-releasing hormone, and brain-derived neuro-trophic factor (BDNF) [30]. CPE KO mice exhibit multiple endocrinopathies leading to obesity, diabetes, and infertility [19]; however, how CPE promotes tumor progression is largely unknown. In this study, we found that CPE upregulation increased the S-phase fraction of CRC cells, thereby promoting cell growth and tumorigenicity. Further investigation indicated that CPE achieved this pro-proliferation effect by modulating p21, p27 expression and mediating cyclin D1 expression at the mRNA level. To date, CPE has been considered to be an enzyme, and not a transcriptional factor or cofactor. This means that CPE cannot initiate transcription of these cell cycle regulators by itself, and therefore further investigation is needed.

In the current study, we found that ectopic expression of CPE dramatically enhanced, whereas silencing of CPE impaired, growth rates of both CRC cell lines. More importantly, soft-agar assay revealed that the anchorage-independent growth of CRC cells lines significantly enhanced upon CPE upregulation and impaired in response to CPE depletion, suggesting that overexpression of CPE promotes, but downregulation of CPE decreased, the tumorigenicity of CRC cells, which are currently under investigation in our laboratory examined with *in vivo* model using CPE-overexpressing and CPE-silenced cells. Meanwhile, Saravana and colleagues have also reported a higher level of CPE in metastatic CRC specimens than primary ones [9], indicating that CPE involves in tumor metastasis. Therefore, it is also worthy to further investigate the correlation and biological role of CPE in CRC metastasis.

In summary, this study showed that CPE was dramatically elevated in CRC cell lines and tissues samples, compared to normal colorectal epithelial cells and matched adjacent normal tissue (ANT), respectively. Further investigations revealed that upregulation of CPE enhanced cell proliferation and tumorigenicity in CRC cells; whereas downregulation impaired cell proliferation and tumorigenicity, and that this was achieved through regulation of the cell cycle regulators p21, p27, and upregulation of cyclin D1. Understanding the precise role of CPE in CRC progression will increase our knowledge of the biological mechanisms of CRC. Suppression of CPE may offer a novel therapeutic strategy for CRC.

## Competing interests

The authors declare that they have no competing interests.

## Authors’ contributions

Conceived and designed the experiments: XCZ GXZ WC. Performed the experiments: XCZ XHL LLL GGW. Analyzed the data: XCZ YCX HFY QLL. Contributed reagents/materials/ analysis tools: XCZ WHL WGH YNH. Wrote the paper: XCZ. All authors read and approved the final manuscript.

## Pre-publication history

The pre-publication history for this paper can be accessed here:

http://www.biomedcentral.com/1471-2407/13/412/prepub

## Supplementary Material

Additional file 1: Figure S1 Expression of CPE splice variants. RT-PCR analyses of the relative expression of CPE splice variants in tumor tissue compared to matched adjacent normal tissue in colorectal cancer. Data were normalized to *GAPDH* control and presented as mean ± SD from three independent experiments. *: *P <* 0.05. Click here for file

## References

[B1] Jemal A Bray F Center MM Ferlay J Ward E Forman D Global cancer statistics CA Cancer J Clin 2011 61 2 69 90 10.3322/caac.20107 21296855

[B2] Eustace K Colorectal cancer Lancet 2005 365 9454 166 10.1016/S0140-6736(05)17707-1 15639299

[B3] Kinzler KW Vogelstein B Lessons from hereditary colorectal cancer Cell 1996 87 2 159 170 10.1016/S0092-8674(00)81333-1 8861899

[B4] Lynch HT de la Chapelle A Hereditary colorectal cancer N Engl J Med 2003 348 10 919 932 10.1056/NEJMra012242 12621137

[B5] Half EE Bresalier RS Clinical management of hereditary colorectal cancer syndromes Curr Opin Gastroenterol 2004 20 1 32 42 10.1097/00001574-200401000-00008 15703618

[B6] Kaneda H Arao T Tanaka K Tamura D Aomatsu K Kudo K Sakai K De Velasco MA Matsumoto K Fujita Y FOXQ1 is overexpressed in colorectal cancer and enhances tumorigenicity and tumor growth Cancer Res 2010 70 5 2053 2063 10.1158/0008-5472.CAN-09-2161 20145154

[B7] Samuels Y Wang Z Bardelli A Silliman N Ptak J Szabo S Yan H Gazdar A Powell SM Riggins GJ High frequency of mutations of the PIK3CA gene in human cancers Science 2004 304 5670 554 10.1126/science.1096502 15016963

[B8] Nosho K Kawasaki T Chan AT Ohnishi M Suemoto Y Kirkner GJ Fuchs CS Ogino S Cyclin D1 is frequently overexpressed in microsatellite unstable colorectal cancer, independent of CpG island methylator phenotype Histopathology 2008 53 5 588 598 10.1111/j.1365-2559.2008.03161.x 18983468 PMC2719983

[B9] Murthy SR Pacak K Loh YP Carboxypeptidase E: elevated expression correlated with tumor growth and metastasis in pheochromocytomas and other cancers Cell Mol Neurobiol 2010 30 8 1377 1381 10.1007/s10571-010-9592-y 21061162 PMC3057539

[B10] Fricker LD Carboxypeptidase E Annu Rev Physiol 1988 50 309 321 10.1146/annurev.ph.50.030188.001521 2897826

[B11] Jean F Basak A Rondeau N Benjannet S Hendy GN Seidah NG Chretien M Lazure C Enzymic characterization of murine and human prohormone convertase-1 (mPC1 and hPC1) expressed in mammalian GH4C1 cells Biochem J 1993 292 Pt 3 891 900 8318017 10.1042/bj2920891 PMC1134198

[B12] Johanning K Juliano MA Juliano L Lazure C Lamango NS Steiner DF Lindberg I Specificity of prohormone convertase 2 on proenkephalin and proenkephalin-related substrates J Biol Chem 1998 273 35 22672 22680 10.1074/jbc.273.35.22672 9712897

[B13] Steiner DF The proprotein convertases Curr Opin Chem Biol 1998 2 1 31 39 10.1016/S1367-5931(98)80033-1 9667917

[B14] Hook VY Loh YP Carboxypeptidase B-like converting enzyme activity in secretory granules of rat pituitary Proc Natl Acad Sci U S A 1984 81 9 2776 2780 10.1073/pnas.81.9.2776 6326144 PMC345153

[B15] Cool DR Normant E Shen F Chen HC Pannell L Zhang Y Loh YP Carboxypeptidase E is a regulated secretory pathway sorting receptor: genetic obliteration leads to endocrine disorders in Cpe(fat) mice Cell 1997 88 1 73 83 10.1016/S0092-8674(00)81860-7 9019408

[B16] Shen FS Loh YP Intracellular misrouting and abnormal secretion of adrenocorticotropin and growth hormone in cpefat mice associated with a carboxypeptidase E mutation Proc Natl Acad Sci U S A 1997 94 10 5314 5319 10.1073/pnas.94.10.5314 9144234 PMC24675

[B17] Chen H Jawahar S Qian Y Duong Q Chan G Parker A Meyer JM Moore KJ Chayen S Gross DJ Missense polymorphism in the human carboxypeptidase E gene alters enzymatic activity Hum Mutat 2001 18 2 120 131 10.1002/humu.1161 11462236

[B18] Naggert JK Fricker LD Varlamov O Nishina PM Rouille Y Steiner DF Carroll RJ Paigen BJ Leiter EH Hyperproinsulinaemia in obese fat/fat mice associated with a carboxypeptidase E mutation which reduces enzyme activity Nat Genet 1995 10 2 135 142 10.1038/ng0695-135 7663508

[B19] Cawley NX Zhou J Hill JM Abebe D Romboz S Yanik T Rodriguiz RM Wetsel WC Loh YP The carboxypeptidase E knockout mouse exhibits endocrinological and behavioral deficits Endocrinology 2004 145 12 5807 5819 10.1210/en.2004-0847 15358678

[B20] Cawley NX Yanik T Woronowicz A Chang W Marini JC Loh YP Obese carboxypeptidase E knockout mice exhibit multiple defects in peptide hormone processing contributing to low bone mineral density Am J Physiol Endocrinol Metab 2010 299 2 E189 E197 20460579 10.1152/ajpendo.00516.2009 PMC2928512

[B21] Pla V Paco S Ghezali G Ciria V Pozas E Ferrer I Aguado F Secretory sorting receptors carboxypeptidase E and secretogranin III in amyloid beta-associated neural degeneration in alzheimer's disease Brain Pathol 2012 23 3 274 284 22998035 10.1111/j.1750-3639.2012.00644.x PMC8028878

[B22] Horing E Harter PN Seznec J Schittenhelm J Buhring HJ Bhattacharyya S von Hattingen E Zachskorn C Mittelbronn M Naumann U The "go or grow" potential of gliomas is linked to the neuropeptide processing enzyme carboxypeptidase E and mediated by metabolic stress Acta Neuropathol 2012 124 1 83 97 10.1007/s00401-011-0940-x 22249620

[B23] Lee TK Murthy SR Cawley NX Dhanvantari S Hewitt SM Lou H Lau T Ma S Huynh T Wesley RA An N-terminal truncated carboxypeptidase E splice isoform induces tumor growth and is a biomarker for predicting future metastasis in human cancers J Clin Invest 2011 121 3 880 892 10.1172/JCI40433 21285511 PMC3049392

[B24] Liu T Papagiannakopoulos T Puskar K Qi S Santiago F Clay W Lao K Lee Y Nelson SF Kornblum HI Detection of a microRNA signal in an in vivo expression set of mRNAs PLoS One 2007 2 8 e804 10.1371/journal.pone.0000804 17726534 PMC1950084

[B25] Krajnik M Schafer M Sobanski P Kowalewski J Bloch-Boguslawska E Zylicz Z Mousa SA Enkephalin, its precursor, processing enzymes, and receptor as part of a local opioid network throughout the respiratory system of lung cancer patients Hum Pathol 2010 41 5 632 642 10.1016/j.humpath.2009.08.025 20040394

[B26] Goodman BK Capone GT Hennessey J Thomas GH Familial tandem duplication of bands q31.1 to q32.3 on chromosome 4 with mild phenotypic effect Am J Med Genet 1997 73 2 119 124 10.1002/(SICI)1096-8628(19971212)73:2<119::AID-AJMG3>3.0.CO;2-P 9409859

[B27] Otsuka T Fujinaka H Imamura M Tanaka Y Hayakawa H Tomizawa S Duplication of chromosome 4q: renal pathology of two siblings Am J Med Genet A 2005 134 3 330 333 15732061 10.1002/ajmg.a.30643

[B28] Angulo MA Castro-Magana M Sherman J Collipp PJ Milson J Trunca C Derenoncourt AN Endocrine abnormalities in a patient with partial trisomy 4q J Med Genet 1984 21 4 303 307 10.1136/jmg.21.4.303 6387124 PMC1049303

[B29] Walboomers JM Jacobs MV Manos MM Bosch FX Kummer JA Shah KV Snijders PJ Peto J Meijer CJ Munoz N Human papillomavirus is a necessary cause of invasive cervical cancer worldwide J Pathol 1999 189 1 12 19 10.1002/(SICI)1096-9896(199909)189:1<12::AID-PATH431>3.0.CO;2-F 10451482

[B30] Lou H Kim SK Zaitsev E Snell CR Lu B Loh YP Sorting and activity-dependent secretion of BDNF require interaction of a specific motif with the sorting receptor carboxypeptidase e Neuron 2005 45 2 245 255 10.1016/j.neuron.2004.12.037 15664176

