# Supplementary figures and images for: Upregulation of CPE promotes cell proliferation and tumorigenicity in colorectal cancer
Source: BMC Cancer. 2013 Sep 5;13:412. doi: 10.1186/1471-2407-13-412 (PMC3844403; doi:10.1186/1471-2407-13-412)

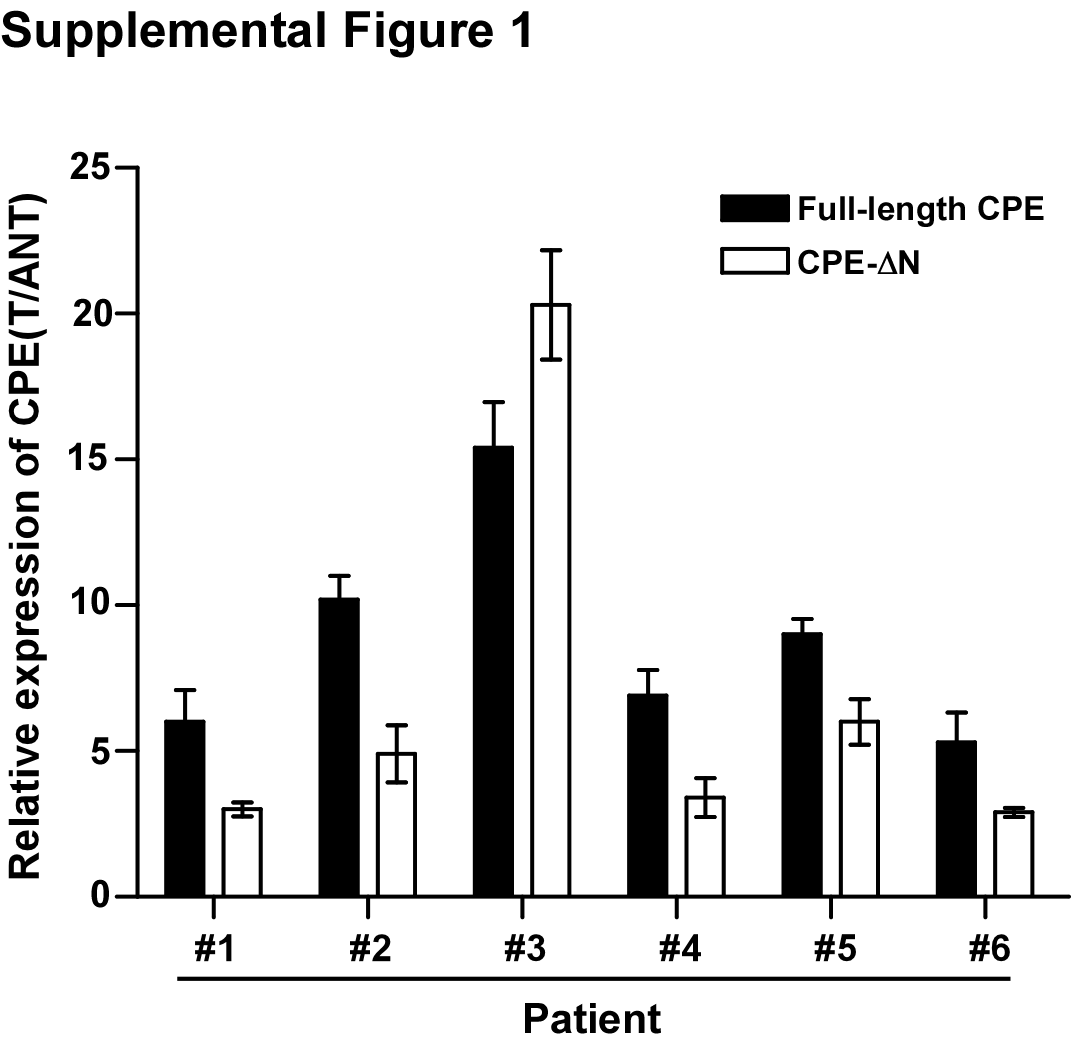

Supplement: Additional file 1: Figure S1 — Expression of CPE splice variants. RT-PCR analyses of the relative expression of CPE splice variants in tumor tissue compared to matched adjacent normal tissue in colorectal cancer. Data were normalized to GAPDH control and presented as mean ± SD from three independent experiments. *: P < 0.05. [file 1471-2407-13-412-S1.tiff]
